# Orexin Neurons Receive Glycinergic Innervations

**DOI:** 10.1371/journal.pone.0025076

**Published:** 2011-09-16

**Authors:** Mari Hondo, Naoki Furutani, Miwako Yamasaki, Masahiko Watanabe, Takeshi Sakurai

**Affiliations:** 1 Department of Molecular Neuroscience and Integrative Physiology, Faculty of Medicine, Kanazawa University, Kanazawa, Ishikawa, Japan; 2 Department of Anatomy and Embryology, Graduate School of Medicine, Hokkaido University, Sapporo, Hokkaido, Japan; Tokyo Metropolitan Institute of Medical Science, Japan

## Abstract

Glycine, a nonessential amino-acid that acts as an inhibitory neurotransmitter in the central nervous system, is currently used as a dietary supplement to improve the quality of sleep, but its mechanism of action is poorly understood. We confirmed the effects of glycine on sleep/wakefulness behavior in mice when administered peripherally. Glycine administration increased non-rapid eye movement (NREM) sleep time and decreased the amount and mean episode duration of wakefulness when administered in the dark period. Since peripheral administration of glycine induced fragmentation of sleep/wakefulness states, which is a characteristic of orexin deficiency, we examined the effects of glycine on orexin neurons. The number of Fos-positive orexin neurons markedly decreased after intraperitoneal administration of glycine to mice. To examine whether glycine acts directly on orexin neurons, we examined the effects of glycine on orexin neurons by patch-clamp electrophysiology. Glycine directly induced hyperpolarization and cessation of firing of orexin neurons. These responses were inhibited by a specific glycine receptor antagonist, strychnine. Triple-labeling immunofluorescent analysis showed close apposition of glycine transporter 2 (GlyT2)-immunoreactive glycinergic fibers onto orexin-immunoreactive neurons. Immunoelectron microscopic analysis revealed that GlyT2-immunoreactive terminals made symmetrical synaptic contacts with somata and dendrites of orexin neurons. Double-labeling immunoelectron microscopy demonstrated that glycine receptor alpha subunits were localized in the postsynaptic membrane of symmetrical inhibitory synapses on orexin neurons. Considering the importance of glycinergic regulation during REM sleep, our observations suggest that glycine injection might affect the activity of orexin neurons, and that glycinergic inhibition of orexin neurons might play a role in physiological sleep regulation.

## Introduction

As the primary inhibitory neurotransmitter in the central nervous system, glycine is widely distributed throughout the brainstem and spinal cord [1], [2], [3]. It also acts as an allosteric modulator of the N-methyl-D-aspartate (NMDA) receptor [4], [5].

Glycine is thought to play important roles in sleep regulation. Especially, medullary glycinergic neurons were shown to inhibit somatic motor neurons during REM sleep [6], [7], [8], [9].

It was also reported that orally administered glycine increased subjective sleep quality without any adverse effect [10]. However, the mechanisms and sites of action of these effects of glycine administration on sleep have remained largely unclear.

In the present study, we confirmed the effect of glycine on sleep/wakefulness states in mice. Notably, glycine not only decreased wakefulness time in the dark period, but also significantly shortened the mean wakefulness duration, suggesting fragmentation of sleep/wakefulness states. Since fragmentation of sleep/wakefulness states is one of the characteristics of orexin-deficiency, we examined whether glycine influences the activity of orexin-producing neurons, which play highly important roles in sleep/wakefulness regulation [11]. We found that peripheral administration of glycine decreased the activity of orexin neurons as examined by Fos-immunoreactivity. We also identified the existence of functional glycine receptors and glycinergic synapses in orexin neurons by electrophysiology, immunofluorescence and immunoelectron microscopy.

## Results

### Sleep/wakefulness States were Influenced by Intraperitoneal Glycine Administration in Mice

**Figure 1:**
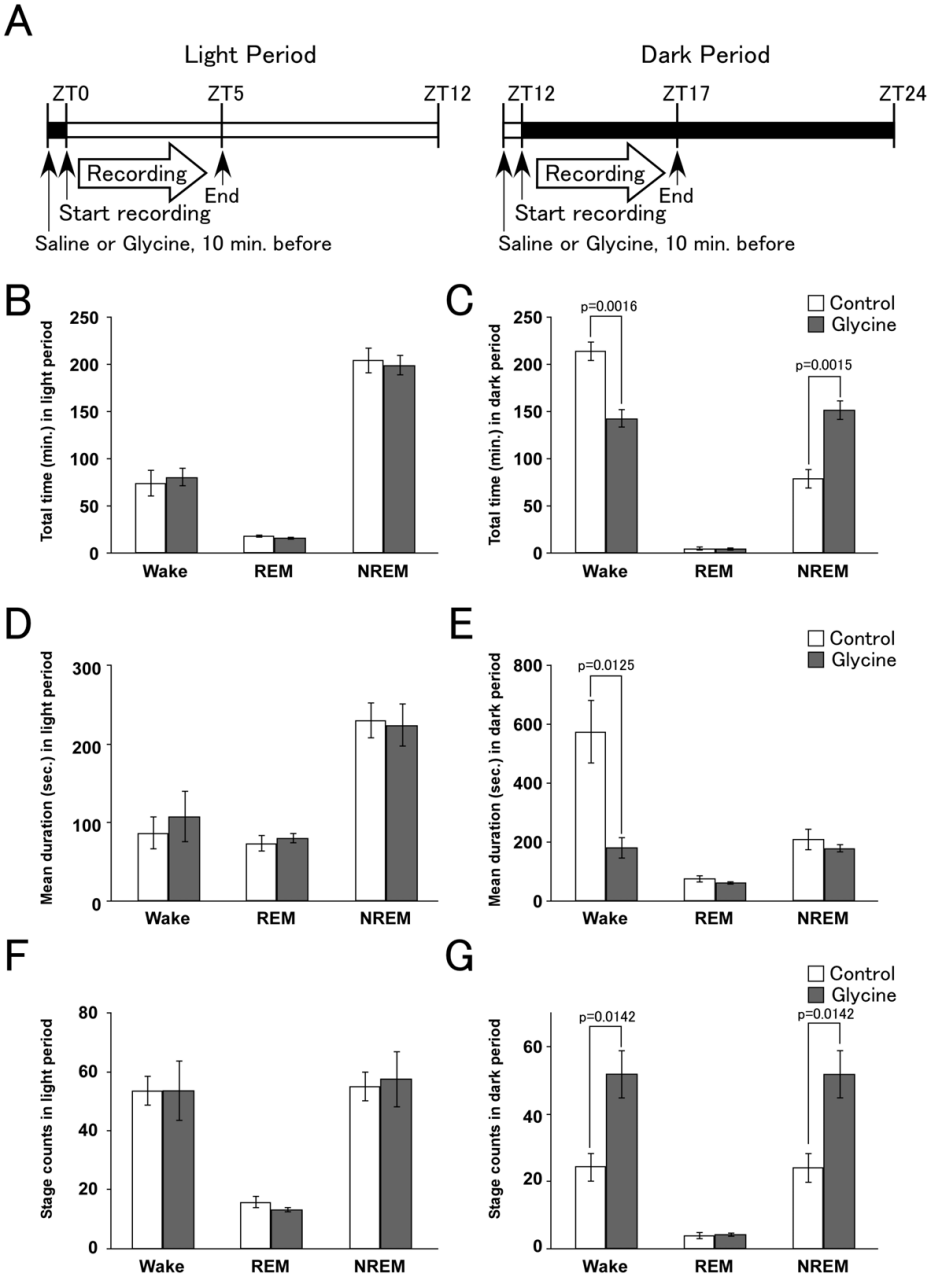
Glycine-induced sleep phenotype during dark period. **A** Protocols for glycine administration in light (left panel) or dark period (right panel). Glycine or saline was injected intraperitoneally 10 min before the start of recording (ZT0 or ZT12). EEG/EMG recordings were performed for the next 5 hours (until ZT5 or ZT17). **B**, **C** Total time (minutes, mean ± SEM) spent in each state in saline- (n = 4, white bar) and glycine-administered mice (n = 4, gray bar), itemized separately for light (B) and dark periods (C). **D**, **E** Episode duration (seconds, mean ± SEM) spent in each state in saline- and glycine-administered mice, in light (D) or dark period (E). **F**, **G** Stage count (count, mean ± SEM) is number of each episode during each period (light; F, dark; G). The glycine-administered group showed a significantly shorter total time and duration of episodes of wakefulness, suggesting fragmentation of sleep/wakefulness states during the dark period. *p<0.015. Graphs summarize the data recorded during the 5 h light/dark period.

Although glycine ingestion is reported to affect subjective sleep quality in humans [10], it is not known whether it actually affects sleep/wakefulness states in mice. Therefore, we first examined the effects of glycine on sleep/wakefulness states in mice. Sleep state patterns in mice were examined by simultaneous EEG/EMG recording after glycine administration. Mice were administered glycine (2 g/kg) or saline intraperitoneally 10 min before light on (ZT0) or off (ZT12), and then subjected to simultaneous EEG/EMG recording for 5 hours (Fig. 1A). In the light period, the pattern of sleep/wakefulness states was not statistically significantly different between the saline- and glycine-administered groups with respect to the total time, mean durations and stage count for all vigilance states (Fig. 1B,D,F).

In contrast, peripheral administration of glycine showed a significant impact on the sleep/wakefulness states of mice in the dark period. There were marked differences between the saline- and glycine-administered groups in the amount of both wakefulness and NREM sleep, duration of wakefulness state, and stage count of both wakefulness and NREM sleep in the dark period (Fig. 1C,E,G). These observations suggest that glycine decreased wakefulness time and increased NREM sleep time in mice when administered at the start of the dark period. Notably, glycine administration resulted in marked shortening of the mean episode duration of wakefulness, accompanied by increased stage count of both wakefulness and NREM sleep (Fig. 1E,G, supplementary Fig. S1). These observations indicate that glycine administration in the dark period decreased the stability of sleep/wakefulness states, and induced sleep/wakefulness fragmentation in mice.

### Activity of Orexin Neurons is Decreased by Intraperitoneal Glycine Administration

**Figure 2:**
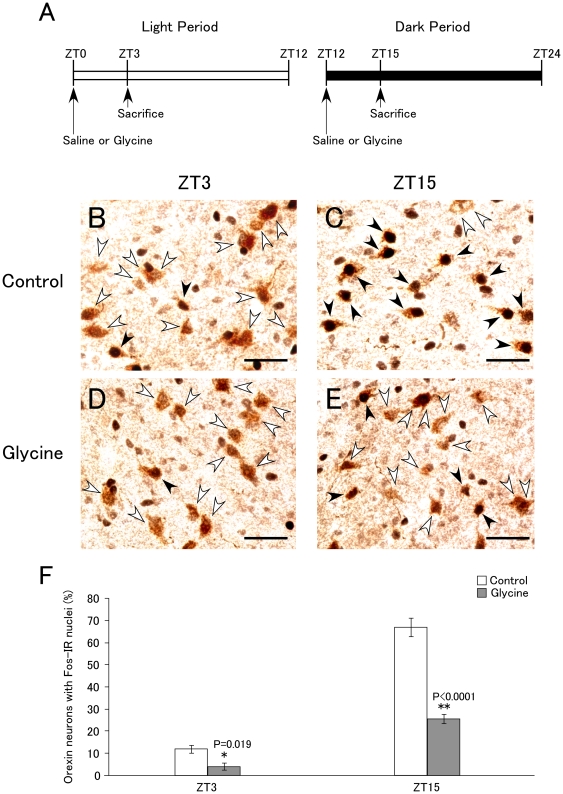
Effects of glycine administration to mice on activity of orexin neurons. **A.** Protocols for glycine administration in light (left panel) or dark period (right panel). Glycine or saline was injected intraperitoneally at ZT0 or ZT12, and then mice were sacrificed at ZT3 or ZT15. **B–E**, Representative immunohistochemical micrographs of double-staining with anti-c-Fos (black) and anti-orexin A (brown) antibody at ZT3 (**B**, control (saline); **D**, glycine (2 g/kg)) and at ZT15 (**C**, control; **E**, glycine). White arrowheads show orexin-immunoreactive cells. Black arrowheads show orexin neurons with Fos-immunoreactivity in their nuclei. Scale bar, 20 µm. **F.** Percentage of c-Fos-expressing orexin neurons at ZT 3 (control; n = 3, glycine; n = 4) and ZT 15 (control; n = 4, glycine; n = 6). Values are mean ± SEM. *p<0.02, **p<0.0001.

Fragmentation of sleep/wakefulness states in the dark period is one of the hallmarks of orexin deficiency or narcolepsy in many mammalian species [11]. Therefore, we next examined the possibility that peripherally-administered glycine influences the activity of orexin neurons. Mice were intraperitoneally administered glycine (2 g/kg B.W.) or saline at ZT0 and ZT12, and then killed 3 hours after administration at ZT3 and ZT15 (Fig. 2A). Brain slices of mice with or without glycine administration were examined by double staining with anti-orexin and anti-Fos antibodies to assess the activity of these orexin neurons. In control mice, the percentage of double-labeled neurons (orexin-positive neurons with Fos-positive nuclei) showed marked diurnal fluctuations, with a lower level of double-labeling at ZT3 (n = 3, 12.1±1.8%, mean ± SEM) and higher level at ZT15 (n = 4, 67.2±4.2%, mean ± SEM) (Fig. 2B,C,F), as reported previously in rats [12]. Glycine administration significantly decreased the percentage of Fos-positive orexin neurons to 4.2±1.5% (n = 4,, mean ± SEM, t_5_ = 3.412, p = 0.019) and 26.2±2.0% (n = 6, mean ± SEM, t_8_ = 9.880, p<0.0001) at ZT3 and ZT15, respectively (Fig. 2D,E,F). Range of orexin cell numbers and c-fos positive cell numbers were shown as supplementary table S1.

### Glycine Inhibits Orexin Neurons *in Vitro*

**Figure 3:**
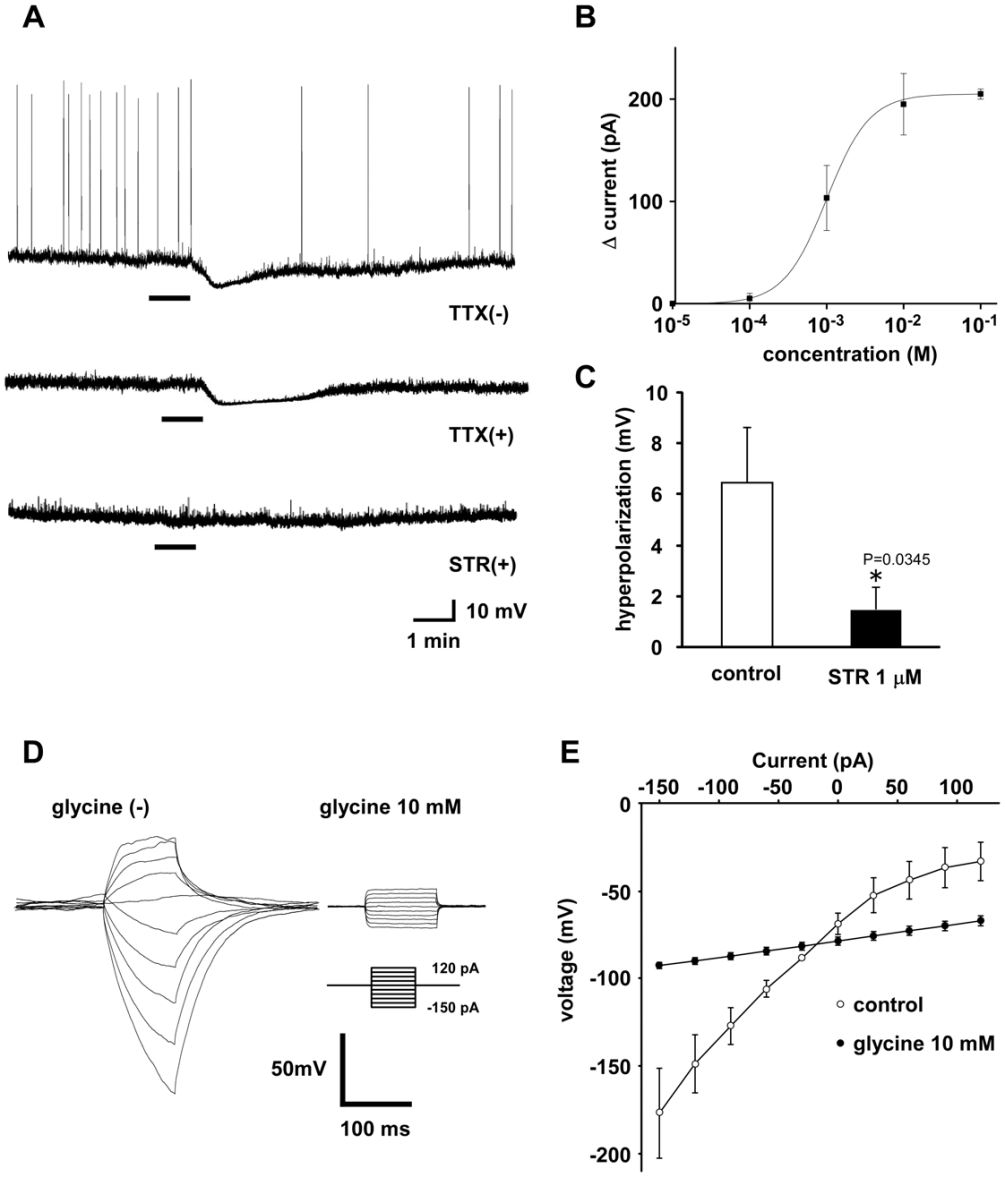
Glycine directly inhibits orexin neurons. **A.** Under whole-cell current-clamp mode, glycine (1 mM) was applied to orexin neurons in normal extracellular solution, in the absence of TTX (upper panel), in the presence of TTX (1 µM) (middle panel) and in the presence of both TTX (1 µM) and strychnine (1 µM) (lower panel). Glycine was applied during the period indicated by the bars. **B.** Concentration dependency of glycine-induced outward currents in orexin neurons clamped at −60 mV. EC_50_ and E_max_ were 10^−3.00±0.17^ M and 205±5 pA, respectively (n = 2–3). **C.** Strychnine (1 µM) significantly inhibited the effect of glycine in the presence of TTX. **D.** Records of membrane potential in response to a series of current steps (from −150 to +120 pA in 30 pA increments) from resting potential (−60 mV) in the absence (left) or presence (right) of glycine (10 mM). **E.** Current-voltage relationship derived from the data in F. The potential at the end of current injection was plotted; control (open circles) and 10 mM glycine (filled circles). Estimated reversal potential was −80.5 mV (n = 3). Values are mean ± SEM.

Since we found that peripheral administration of glycine decreased the activity of orexin neurons, we next examined whether glycine directly inhibits orexin neurons. We carried out whole-cell current-clamp recordings on acute slice preparations of *orexin/EGFP* transgenic mice [13]. Under whole-cell current clamp mode, glycine (1 mM) bath application hyperpolarized orexin neurons, with a decrease in firing frequency (Fig. 3A). Hyperpolarization was also observed in the presence of TTX (Fig. 3A), suggesting that glycine directly inhibits orexin neurons. The response peaked 30–60 sec after application of glycine, and the membrane potential returned to the basal level 2–3 min after washout. The majority of GFP-positive neurons tested were hyperpolarized by glycine (89%, 33 out of 37), and a few orexin neurons showed no response (11%, 4 out of 37). Figure 3B demonstrates that glycine-induced inward currents recorded in a voltage-clamp at −60 mV were concentration-dependent; EC_50_ and maximum effect (E_max_) were 10^−3.00±0.17^ M and 205±5 pA, respectively (n = 2–3). Additionally, glycine (1 mM)-induced hyperpolarization was significantly inhibited by strychnine (1 µM), suggesting that the inhibitory effect is dependent on the ionotropic glycine receptor (GlyR) (Fig. 3C). The reversal potential estimated from the I–V relationship was −80.6±2.4 mV (n = 3), which is near the theoretically obtained value of this parameter for Cl^−^ (−77.5 mV at 25°C) (Fig. 3D, E). This value is similar to that of the theoretical Cl^−^-selective ion channel calculated by the Nernst equation, in agreement with known biophysical properties of GlyR Cl^−^ channels.

### Existence of Glycinergic Synapses in Orexin Neurons

**Figure 4:**
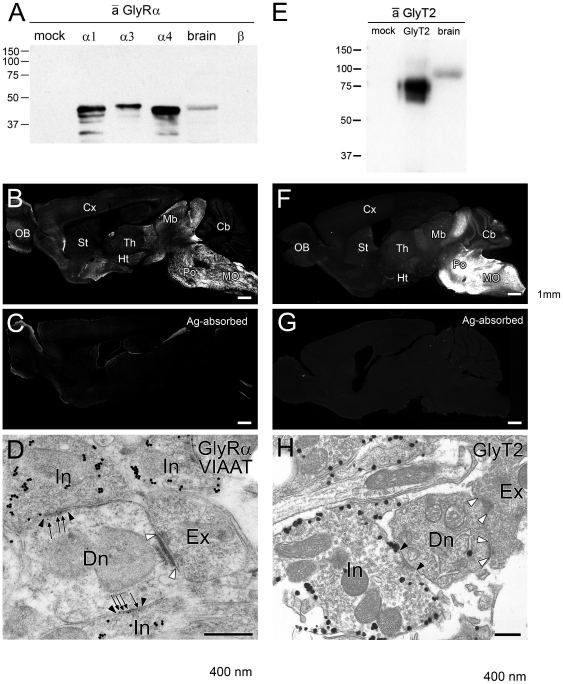
Specificity of GlyRα and GlyT2 antibodies. **A**, **E.** Immunoblotting using GlyRα and GlyT2 (E) antibodies. Each lane was loaded with HEK293 cell lysates transfected with expression plasmid only (mock), transfected with expression plasmid encoding GlyRα1, GlyRα3, GlyRα4, GlyRβ, or GlyT2, and adult mouse brain homogenates. Because of low viability, HEK293 cell lysate of GlyRα2-transfected cells was omitted. Identical patterns of immunoblot labeling were repeatedly confirmed in three independent experiments. The position of protein size markers (kDa) is shown on the left. **B**, **C**, **F**, **G.** Immunofluorescence with use of GlyRα (B, C) or GlyT2 (F, G) in parasagittal brain sections. Note that preincubation of antibodies with antigen protein (C, G) almost completely abolished immunostaining. **D.** Double-labeling postembedding immunogold for GlyRα (10 nm) and VIAAT (15 nm) in the facial nucleus. Note that GlyRα localizes to a symmetrical synapse (black arrowheads) made with a VIAAT-positive inhibitory terminal (In), but not to an asymmetrical synapse (white arrowheads) made with a VIAAT-negative excitatory terminal (Ex). Arrows indicate immunogold particles for GlyRα. **H.** Preembedding silver-enhanced immunoelectron microscopy of facial nucleus. Metal particles for GlyT2 are preferentially distributed on the cell membrane of presynaptic inhibitory terminals (In), which contain flat synaptic vesicles and make symmetrical synapses with dendritic shafts (Dn). OB, olfactory bulb; Cx, cerebral cortex; St, striatum; Th, thalamus; Ht, hypothalamus; Mb, midbrain; Po, pons; Cb, cerebellum; MO, medulla oblongata; Ex, excitatory terminal; In, inhibitory terminal; Dn, dendrite. Scale bars, B, C, F, G; 1 mm; D, H, 400 nm.

The responsiveness of orexin neurons to glycine suggests the possibility that these neurons are physiologically regulated by glycinergic neurotransmission. To address this possibility, we produced specific antibodies against bacterial fusion proteins encoding the glycine receptor common to all four α subunits (GlyRα) and plasmalemmal glycine transporter 2 (GlyT2). By immunoblotting, GlyRα antibody recognized protein bands at around 45 kDa in both HEK293 cell lysates expressing mouse GlyRα subunits and adult mouse brain, but not in HEK293 cell lysates transfected with plasmid vector only or expressing mouse GlyRβ subunit (Fig. 4A). GlyT2 antibody selectively recognized a broad protein band at around 75 kDa in HEK293 cell lysates expressing mouse GlyT2, and at 90 kDa in adult mouse brain (Fig. 4A). On immunofluorescence, both antibodies mainly labeled the lower brainstem (Fig. 4B, F), while those preabsorbed with antigens yielded blank labeling (Fig. 4C, G). Immunoreactivity of these antibodies was further characterized by immunoelecrtron microscopy of the facial nucleus. Double-labeling with postembedding immunogold for GlyRα and vesicular inhibitory amino acid transporter (VIAAT) revealed that the postsynaptic membrane was consistently labeled for GlyRα at symmetrical synapses (95%; 20 out of 21 synapses), but not asymmetrical synapses (0%; 0 out of 15 synapses) (Fig. 4D). The mean number of immunogold particles per contact site was 4.9+4.7 (mean + SD) in dendites of facial nucleus neurons. Preembedding immunoelectron microscopy also showed that immunoparticles for GlyT2 were selectively associated with presynaptic terminals forming symmetrical synapses (100%, 10 out of 10 terminals), but not asymmetrical synapses (0%, 0 out of 10 terminals) (Fig. 4H). As symmetrical and asymmetrical synapses are considered to mostly, if not totally, represent inhibitory or excitatory synapses, respectively, these results indicate the specificity of the produced GlyRα and GlyT2 antibodies and their usefulness to identify glycinergic synapses.

**Figure 5:**
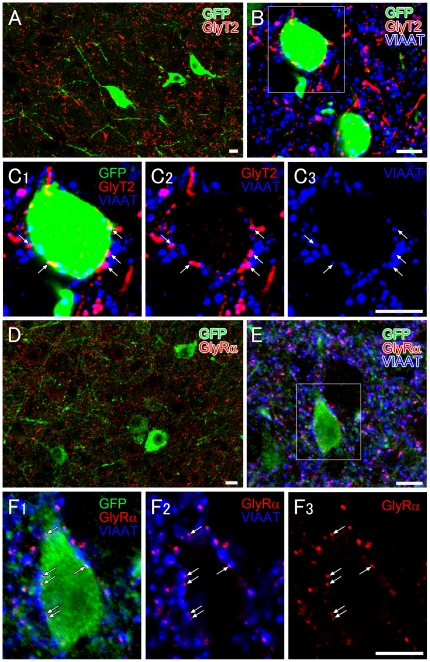
Immunofluorescence showing glycinergic innervations to orexin neurons. **A.** Double immunofluorescence showing distribution of GlyT2-positive glycinergic varicose fibers (red) and GFP-positive orexin neurons (green) in the LHA. **B**, **C.** Triple immunofluorescence showing that orexin neurons (GFP, green) are associated with GlyT2-positive (red)/VIAAT-positive (blue) varicosities (arrows). **D.** Double immunofluorescence showing the distribution of GlyRα- (red) and GFP-positive orexin neurons (green) in the LHA. **E**, **F.** Orexin neurons (green) display numerous VIAAT-positive inhibitory terminals (blue), some of which are associated with GlyRα immunoreactivity (red) on the surface of orexin neurons. Scale bars, 10 µm.

**Figure 6 pone-0025076-g006:**
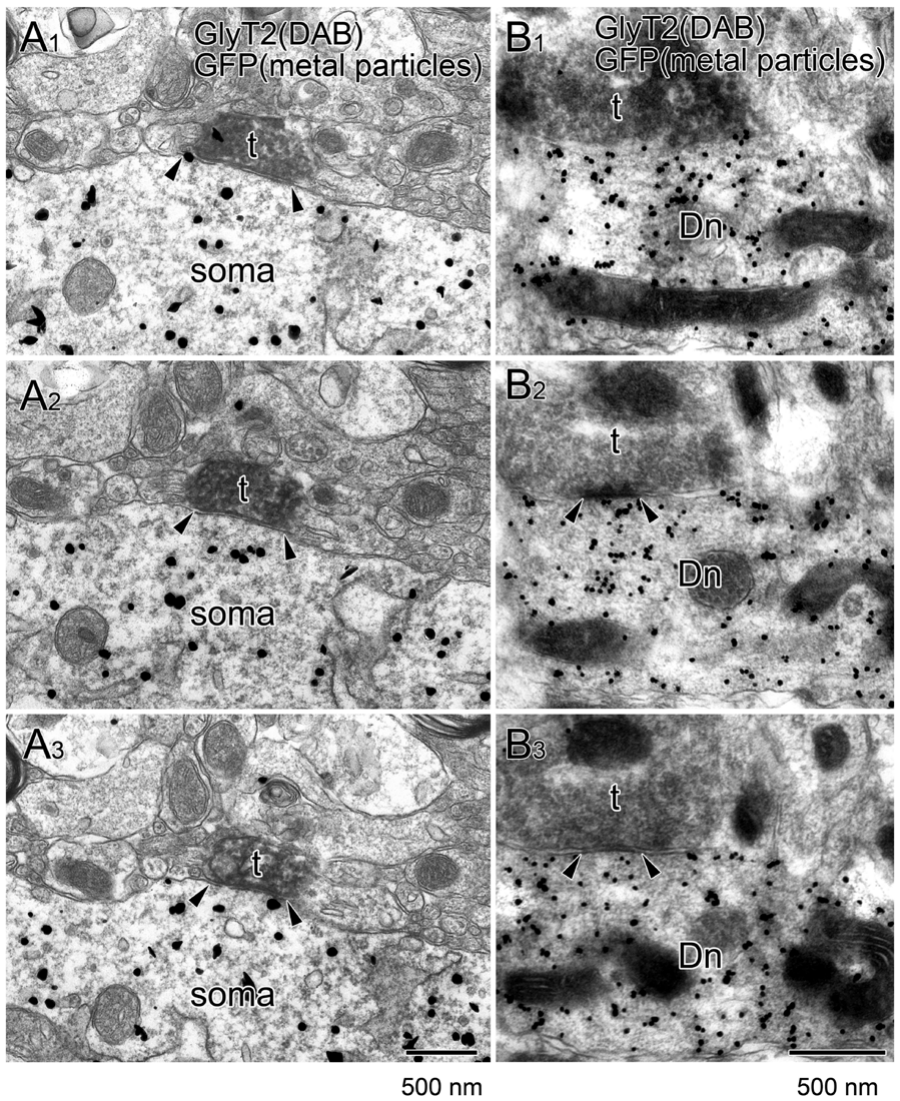
Immunoelectron microscopy showing glycinergic synapse formation on orexin neurons. Consecutive images from double-labeling preembedding immunoelectron microscopy for GFP (silver-intensified immunogold) and GlyT2 (immunoperoxidase). Note that a GlyT2-positive terminal (filled with diffuse DAB precipitates) makes a symmetrical synaptic junction (arrowheads) onto the soma (**A**) and dendritic shaft (**B**) of a GFP-positive orexin neuron. Scale bar, 500 nm.

Although the overall immunofluorescence intensity was much lower in the hypothalamus than in the lower brainstem (Fig. 4B, F), punctate labeling of GlyT2 and GlyRα did exist around GFP-positive orexin neurons in the lateral hypothalamic area (LHA) (Fig. 5A, D). At higher magnification, almost all somata [95% (36 out of 38)] and proximal dendrites [95% (19 out of 20)] of GFP-positive orexin neurons were associated with GlyT2-positive varicosities (Fig. 5B, C). Since glycinergic terminals require VIAAT for glycine filling into synaptic vesicles, we further examined if GlyT2-positive varicosities on orexin neurons co-expressed VIAAT (Fig. 5B, C). Indeed, VIAAT coexisted in most GlyT2-positive varicosities associated with somata [85% (49 out of 58)] or proximal dendrites [84% (38 out of 45)], suggesting the formation of glycinergic synapses. To confirm this at the electron microscopic level, we employed double-labeling immunoelectron microscopy for GlyT2 (dark precipitates of DAB) and GFP (metal particles) (Fig. 6). Analysis of serial ultrathin sections revealed that GlyT2-positive glycinergic varicosities occasionally contacted somata (Fig. 6A) and proximal dendrites (Fig. 6B) of GFP-positive orexin neurons. At contact sites, both sides of the apposed membranes had electron-dense material and were spaced with a cleft of uniform width, thus being judged to be a symmetrical synaptic junction (Fig. 6). For quantitative analysis, we randomly selected GFP-positive profiles of somata and dendrites, and examined if they had such synaptic contacts with GlyT2-positive varicosities. On six consecutive sections through each profile, we found that 31% (4 out of 13) of GFP-positive somata and 23% (13 out of 65) of GFP-positive dendrites had synaptic contact with GlyT2-positive varicosities, all of which were judged to be symmetrical synapses. These results confirm the existence of symmetrical synaptic junctions between glycinergic terminals and GFP-positive orexin neurons.

**Figure 7:**
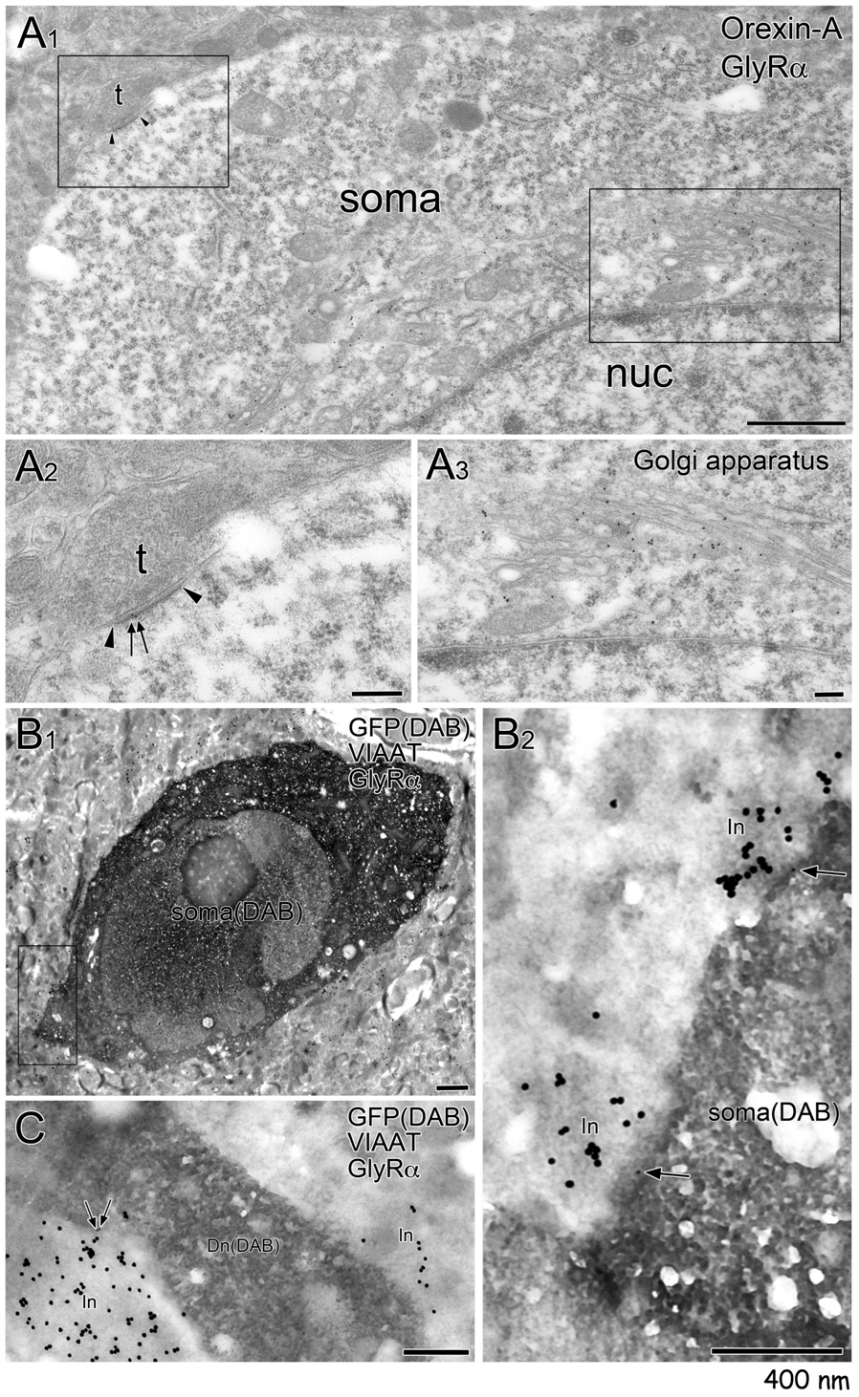
Postembedding immunogold showing glycine receptor localization at symmetrical synapses. **A.** Double-labeling postembedding immunogold for GlyRα (small particles) and orexin A (large particles) shows that an orexin neuron, whose Golgi apparatus is heavily labeled with immunogold for orexin-A (A_3_), expresses GlyR at a symmetrical synapse (black arrowheads, A_2_). **B**,**C.** Triple-labeling immunoelectron microscopy for GFP (dark precipiates by preembedding immunoperoxidase), GlyRα (10 nm, postembedding immunogold) and VIAAT (20 nm, postembedding immunogold). GlyRα is localized to contact sites made between VIAAT-positive inhibitory terminals (In) and the soma (B_2_) or dendritic shaft (Dn, C) of GFP-positive orexin neurons. Scale bars, 1 µm in B_1_, 400 nm in A,B_2_,C.

We next examined if glycine receptors accumulated at such symmetrical synaptic junctions. GlyRα-positive puncta were frequently detected on the surface of GFP-positive orexin neurons, and were tightly paired with VIAAT-positive varicosities (Fig. 5E, F). Such co-localization was observed in 71% (12 out of 17) and 82% (9 out of 11) of somata and proximal dendrites, respectively, of GFP-positive neurons. By double-labeling postembedding immunogold for GlyRα (10 nm in diameter) and orexin A (15 nm), we found immunogold particles for GlyRα at symmetrical synapses on neuronal somata, whose Golgi apparatus was heavily labeled for orexin A (Fig. 7A). For quantitative analysis, we finally employed triple-labeling immunoelectron microscopy, in which postembedding immunogold for VIAAT (20 nm) and GlyRα (10 nm) was applied to LHA sections that had been subjected to immunoperoxidase for GFP (dark precipitates of DAB). Somata and proximal dendrites of GFP-positive neurons showed frequent contacts with VIAAT-positive terminals (Fig. 7B,C). Occasionally, their contact sites were labeled for GlyRα; immunogold particles for GlyRα were detected in 13% (3 out of 23) and 26% (12 out of 46) of contact sites in GFP-positive somata and dendrites, respectively. The mean number of immunogold particles per contact site was 0.13±0.34 in somata and 0.52±0.96 in dendites of GFP-positive orexin neurons. Taken together, these observations indicate that the glycine receptor is expressed in glycinergic synapses in orexin neurons.

## Discussion

Glycine, an inhibitory neurotransmitter, has been implicated in the physiological regulation of sleep. Particularly, glycinergic neurons in the brainstem play important roles in inhibition of alpha-motor neurons in the spinal cord during REM sleep [9]. A glycine receptor antagonist, strychnine, was also reported to reduce the inhibition of sensory inflow via the dorsal spinocerebellar tract during REM sleep [14], [15], [16]. Orally-administered glycine was reported to have a beneficial effect on sleep in humans [10]. This suggests that peripherally-administered glycine might act on neuronal systems that play important roles in sleep regulation.

Orexins are neuropeptides, produced by neurons in the LHA, which send projections to all over the central nervous system except the cerebellum. The finding that orexin deficiency causes narcolepsy in humans and animals highlights that these hypothalamic neuropeptides play a critical role in regulating sleep/wakefulness states [11].

In the present study, we first confirmed the effect of glycine administration on sleep/wakefulness states in mice. We found that peripheral administration of glycine significantly decreased wakefulness time, and caused sleep/wakefulness fragmentation in the dark period (Fig. 1). Therefore, we investigated the effects of glycine on orexin neurons, because orexin plays highly important roles in maintenance of wakefulness, and deficiency of orexin signaling has been shown to result in sleep/wakefulness fragmentation, a characteristic of narcolepsy [11]. By immunohistochemical study, we found that the number of Fos-positive orexin neurons in glycine-treated mice was significantly lower at both ZT3 and ZT15 as compared with that in saline-treated mice at the same times (Fig. 2).

The percentage of Fos-positive orexin neurons has been shown to correlate positively with the amount of wakefulness and negatively with the amounts of NREM and REM sleep [12], [17]. We recently found that specific pharmacogenetic inhibition of orexin neurons during the active period decreased wakefulness time and increased NREM sleep time in the dark period [18]. These observations suggest the possibility that ingested glycine might affect sleep states through inhibiting orexin neuronal activity, although it remains unknown whether peripherally-administered glycine can act on orexin neurons in the LHA, or it indirectly affects the activity of these neurons. For example, extracellular serotonin levels are reported to be increased after oral administration of glycine [19]. Since we found serotonin directly inhibits orexin neurons [20], glycine administration might partly inhibit orexin neurons through serotonin.

We found that glycine administration decreases Fos-expression in orexin neurons in both the light and dark periods. However, we only observed a significant effect of glycine administration on sleep/wakefulness states in mice in the dark period. This observation is consistent with the previous observations that orexin-deficient mice showed fragmentation of sleep/wakefulness states only during the dark period [12], [21], [22]. Numbers of orexin neurons after glycine administration were not different from that of after saline administration as shown in supplementary table S1. This suggests that glycine did not decrease number of orexin neurons, and glycine does not show harmful effects on these cells.

We found that glycine can directly inhibit orexin neurons in vitro. In the whole-cell current-clamp, most orexin neurons tested in this experiment were potently hyperpolarized by glycine in a concentration-dependent manner (Fig. 3). The response was blocked by a specific glycine receptor antagonist, strychnine. Karnani et al. also reported similar results recently [23]. Furthermore, we have demonstrated the expression of GlyR at glycinergic synapses in orexin neurons (Figs. 5, 6). These observations provide a functional and molecular basis for physiological glycinergic regulation of orexin neurons. However, this does not necessarily mean that peripherally administered glycine directly inhibits orexin neurons. The humoral and/or neuronal pathways that mediate glycine-induced inhibition of orexin neurons should be further confirmed in future studies.

It should also be noted that glycine might affect arousal not only by inhibiting orexin neurons. Systemic glycine might increase sleep duration also through action on other arousal controlling regions in the brain, such as monoaminergic/cholinergic neurons in the brain stem. The contribution of orexin neurons to this effect should be confirmed by studies using spatially restricted knock-out GlyR in mice.

In any case, the existence of GlyR in orexin neurons suggests the possibility that orexin neurons are physiologically regulated by glycinergic neurotransmission. Indeed, we identified glycinergic innervations that made synapses onto orexin neurons by means of immunofluorescent staining and immunoelectron microscopy (Figs. 5–7). This observation suggests the possible existence of physiological glycinergic regulation of orexin neurons.

Glycinergic neurons in the brainstem were shown to play important roles in inhibition of somatic motor neurons in the spinal cord during REM sleep to evoke REM-atonia. The activity of orexin neurons was shown to be decreased during both NREM and REM sleep [24], [25], [26]. These observations suggest an intriguing possibility that glycinergic innervations to orexin neurons might play a role to inhibit the discharge of orexin neurons during REM sleep, although the origin of glycinergic fibers innervating orexin neurons in the LHA remains unknown. Further study using selective deletion of glycine receptor gene(s) in orexin neurons, and antero- and/or retrograde tracer studies would be necessary to evaluate this hypothesis.

Many factors have been identified to regulate orexin neurons, suggesting that orexin neurons undergo regulation by a number of physiological factors [11]. In the present study, we identified that glycine also affects the activity of orexin neurons, when administered peripherally. Furthermore, we identified synaptic connections between glycinergic fibers and orexin neurons, suggesting the possibility that orexin neurons are also physiologically regulated by glycinergic neurotransmission.

This study adds further clues to understand the precise regulatory mechanism of orexin neurons.

## Materials and Methods

### Animals

All experimental procedures involving animals were conducted with the approval of the Kanazawa University Animal Care and Use Committee and the Hokkaido University Animal Welfare Committee, and were in accordance with NIH guidelines. All efforts were made to minimize animal suffering and to limit the number of animals used. Mice were housed under controlled lighting (12 h light-dark cycle; light on at 8:45 a.m., off at 8:45 p.m.) and temperature conditions. Food and water were available *ad libitum*.

### Drugs and method of administration

For in vivo experiments, glycine (2 g/kg saline) or saline alone was injected intraperitoneally (ip) into adult male mice C57BL/6J (10–12 weeks old, weight 20–25 g; Charles River Laboratories, Kanagawa, Japan), at zeitgeber time (ZT) ZT0 or ZT12; ZT0 is morning light onset. The dose of glycine was selected baed on a previous report [19]. Three hours after administration, at ZT3 and ZT15, mice were anesthetized with sodium pentobarbital (50 mg/kg, ip). The drugs used for electrophysiological studies were tetrodotoxin (TTX) (Wako, Osaka, Japan), glycine (Wako), and the glycine receptor antagonist, strychnine (Sigma-Aldrich Corp., St. Louis, MO), which were dissolved in extracellular solution.

### Sleep recording

Male C57BL/6J mice (10–12 weeks old, 20–25 g at the time of surgery) were prepared for chronic monitoring of EEG/EMG signals using a lightweight implant and cabling procedure. Full details of this technique have been published previously [27]. Immediately after surgery, mice were housed singly for a recovery period of one week. Then, EEG/EMG recording for two consecutive 24-h periods, beginning at light on at 08:45 and light off at 20:45, was performed. Glycine was administered (ip) 10 min before light on (ZT0) or off (ZT12) to mice, before they were subjected to sleep recording for 5 hours.

### Electrophysiological recordings

*Orexin/EGFP* mice were used for whole cell recordings [13]. The slices were transferred to a recording chamber (RC-27L, Warner Instrument Corp., CT, USA) at room temperature on a fluorescence microscope stage (BX51WI, Olympus, Tokyo, Japan). Neurons that showed GFP fluorescence were used for patch-clamp recordings. The fluorescence microscope was equipped with an infrared camera (C-3077, Hamamatsu Photonics, Hamamatsu, Japan) for infrared differential interference contrast (IR-DIC) imaging and a CCD camera (JK-TU53H, Olympus) for fluorescent imaging. Each image was displayed separately on a monitor. Recordings were carried out with an Axopatch 700B amplifier (Axon Instruments, Foster City, CA) using a borosilicate pipette (GC150-10, Harvard Apparatus, Holliston, MA) prepared with a micropipette puller (P-97, Sutter Instruments, Pangbourne, UK) and filled with intracellular solution (4–10 MΩ), consisting of (mM): 125 K-gluconate, 5 KCl, 1 MgCl_2_, 10 HEPES, 1.1 EGTA-Na_3_, 5 MgATP, 0.5 Na_2_GTP, pH 7.3 with KOH. Osmolarity of the solution was checked with a vapor pressure osmometer (model 5520, Wescor, Logan, UT). The osmolarity of the internal and external solutions was 280–290 and 320–330 mOsm/l, respectively. The liquid junction potential of the patch pipette and perfused extracellular solution was estimated to be −16.2 mV and was applied to the data. The recording pipette was under positive pressure while it was advanced toward individual cells in the slice. A tight seal of 0.5–1.0 GΩ was made by applying negative pressure. The membrane patch was then ruptured by suction. The series resistance during recording was 10–25 MΩ and was compensated. The reference electrode was an Ag-AgCl pellet immersed in bath solution. During recordings, cells were superfused with extracellular solution at a rate of 1.0–2.0 ml/min using a peristaltic pump (K.T. Lab, Japan) at RT. We adjusted the resting membrane potential of cells to around −60 mV by injecting current of −20 to 40 pA before application of glycine.

### Antibodies

Primary antibodies used in this study were guinea-pig anti-orexin antibody (1∶2000) [28], rabbit anti-Fos antibody (1∶20,000) (Ab-5, Calbiochem, Darmstadt, Germany), rabbit anti-green fluorescent protein (GFP) [29], rabbit anti-vesicular inhibitory amino acid transporter (VIAAT) [30], guinea pig anti-glycine receptor α-subunits (GlyRα), guinea pig and rabbit anti-glycine transporter 2 (GlyT2), and rabbit anti-orexin-A antibodies. GlyRα and GlyT2 antibodies were produced against glutathione S-transferase (GST) fusion proteins containing 105–134 amino acid residues of mouse GlyRα1 (GeneBank, NM_020492), which is common to all four GlyRα, and 1–30 residues of mouse GlyT2 (AF411042). Orexin A antibody was produced against Keyhole limpet hemocyanin-conjugated synthetic peptide of mouse orexin-A (54–65 residues, NM_010410). Specific antibodies were affinity-purified using GST-free or carrier-free peptides. The specificity of GlyRα and GlyT2 antibodies was tested by immunoblotting using HEK cell lysates transfected with pTracer (Invitrogen) and pEF-BOS [31] mammalian expression vectors encoding GlyRα, GlyRβ,or GlyT2, respectively.

### Immunohistochemistry

Mice were fixed transcardially with 4% paraformaldehyde in 0.1 M sodium phosphate buffer (pH 7.2, PB) followed by 4 hr postfixation at 4°C. For Fos immunostaining, sections of fixed brains were cut into 40-µm-thick coronal sections. For orexin and c-Fos double staining, sections were incubated with anti-orexin antibody and anti-Fos antibody for 24 hr at 4°C. Sections were then incubated with biotinylated goat anti-rabbit IgG (1∶1,000; Vector Laboratories, Burlingame, CA, USA) for 1 hr at room temperature. Tissue was then reacted with avidin-biotin complex (Vectastain ABC Elite kit; Vector Laboratories, Burlingame, CA, USA) for 1 hr, and Fos-immunoreactive (IR) nuclei were visualized by reaction with 3,3′-diaminobenzidine hydrochloride (DAB) solution containing 0.003% H_2_O_2_ and 0.05% nickel chloride to obtain a black reaction product. After c-Fos staining, the sections were incubated with biotinylated-goat anti-guinea-pig IgG antibody (1∶1000; Vector Laboratories) for 1 hr at room temperature. The sections were next incubated for 30 min with Vectastain ABC Elite reagents, rinsed, and stained in 0.05% DAB without 0.05% nickel chloride to obtain a cytoplasmic brown reaction product. For immunofluorescent staining, *orexin/EGFP* mouse brain sections were first subjected to pepsin pretreatment for antigen exposure, i.e., incubation in 1 mg/ml pepsin (DAKO, Carpinteria, CA) in 0.2 N HCl for 2–3 min at 37°C. Sections were incubated successively with 10% normal donkey serum for 20 min, primary antibodies to GFP, GlyT2 or GlyRα, and VIAAT (1 µg/ml for each) overnight, and a mixture of Alexa 488-, Cy3- or Cy5-labeled species-specific secondary antibodies for 2 hr at a dilution of 1∶200 (Invitrogen; Jackson ImmunoResearch). Images were obtained with a confocal laser scanning microscope FV1000 (Olympus). Z-stacks 6 to 8 µm thick composed of 1-µm-thick optical sections were obtained with a 60× oil-immersion lens.

### Cell counts

A single examiner, who was blinded to treatment conditions, performed all counts using a microscope (AX-70, Olympus Optical). Fos-IR nuclei, orexin-IR neurons, and double-labeled neurons were counted on both sides of the brain in nine consecutive sections that covered the entire hypothalamus. The percentage of double-labeled cells for each animal (double-labeled neurons/orexin-IR neurons) was calculated as a measure of orexin neuron activity.

### Immunoelectron microscopy

*Orexin/EGFP* mice were fixed transcardially with 4% paraformaldehyde in PB (pH 7.2) for 10 min, and postfixed at 4°C for 4 hr. For postembedding immunogold, microslicer sections (400 µm) were made using a microslicer (VT1000S, Leica). For triple-labeling postembedding immunogold, microslicer sections were first subjected to immunoperoxidase of GFP labeling; sections were incubated successively with 10% normal donkey serum for 20 min, and goat anti-GFP overnight at room temperature. Sections were further incubated with biotinylated donkey anti-goat IgG (Jackson ImmunoResearch) for 2 hr, and streptavidin-peroxidase complex for 1 hr (Nichirei). Immunoreaction was visualized with DAB. Sections with or without preceding immunoperoxidase were cryoprotected with 30% glycerol in PB, and frozen rapidly with liquid propane in the EM CPC unit (Leica Microsystems). Frozen sections were immersed in 0.5% uranyl acetate in methanol at −90°C in the AFS freeze-substitution unit (Leica Microsystems), infiltrated at −45°C with Lowicryl HM-20 resin (Chemische Werke Lowi), and polymerized with UV light. Ultrathin sections on nickel grids were etched with saturated sodium ethanolate solution for 1–5 s, and treated successively with blocking solution [2% normal goat serum (Nichirei) in 0.03% Triton X-100 in Tris-buffered saline (TBST; pH 7.4)] for 20 min, anti-GlyRα (20 µg/ml) diluted with the same blocking solution overnight, and colloidal gold (10 nm)-conjugated goat anti-guinea-pig IgG (1∶100, British BioCell International) for 2 hr. After washing with TBST, grids were incubated with 2% normal guinea pig serum for 20 min in TTBST, rabbit anti-VIAAT or anti-orexin A (20 µg/ml), diluted with the same blocking solution overnight, followed by colloidal gold (15 nm)-conjugated goat anti-rabbit IgG (1∶100, British BioCell International) for 2 hr. Finally, grids were fixed with 2% glutaraldehyde in PB for 15 min and 1% OsO4 for 20 min, and stained with 2% uranyl acetate for 10 min and Reynold's lead citrate solution for 1 min.

For double-labeling preembedding immunoelectron microscopy, microslicer sections were incubated in 5% bovine serum albumin (BSA)/0.02% saponin/PBS for 30 min and then with a mixture of rabbit anti-GFP and guinea pig anti-GlyT2 (1 µg/ml each) diluted with 1% BSA/0.004% saponin/PBS overnight, and with 1.4 nm gold particles-conjugated goat anti-rabbit IgG (Nanogold; Nanoprobes) for 2 hr. Immunogold particles were intensified with a silver enhancement kit (R-Gent silver enhancement kit, Aurion). Sections were further incubated with biotinylated donkey anti-guinea pig IgG (Jackson ImmunoResearch) for 2 hr, and streptavidin-peroxidase complex for 30 min (Nichirei). Immunoreaction was visualized with DAB, and then sections were treated with 1% osmium tetroxide for 15 min, stained with 2% uranyl acetate for 30 min, dehydrated, and embedded in Epon 812. Photographs were taken with an H-7100 electron microscope (Hitachi).

### Statistical analysis

Data were analyzed by unpaired Student's t-test, using the Stat View 4.5 software package (Abacus Concepts, Berkeley, CA). A value of p<0.05 was considered statistically significant.

## Supporting Information

Figure S1 Representative 5 h dark/light period hypnograms for mice after saline and glycine administration. The shaded areas represent the dark period. W, awake; NR, non-rapid eye movement (REM) sleep; R, REM sleep. Glycine administered mice showed fragmentation of sleep/wake states in dark phase.
(TIF)

Table S1 Numbers of orexin neurons with or without Fos immunoreactivity in nuclei after glycine or saline administration. Mice were administered with glycine or saline at ZT0 or ZT12, and sacrificed for immunostaining at ZT3 or ZT15, respectively.
(TIF)
