# Supplementary figures and images for: Orexin Neurons Receive Glycinergic Innervations
Source: PLoS One. 2011 Sep 16;6(9):e25076. doi: 10.1371/journal.pone.0025076 (PMC3174993; doi:10.1371/journal.pone.0025076)

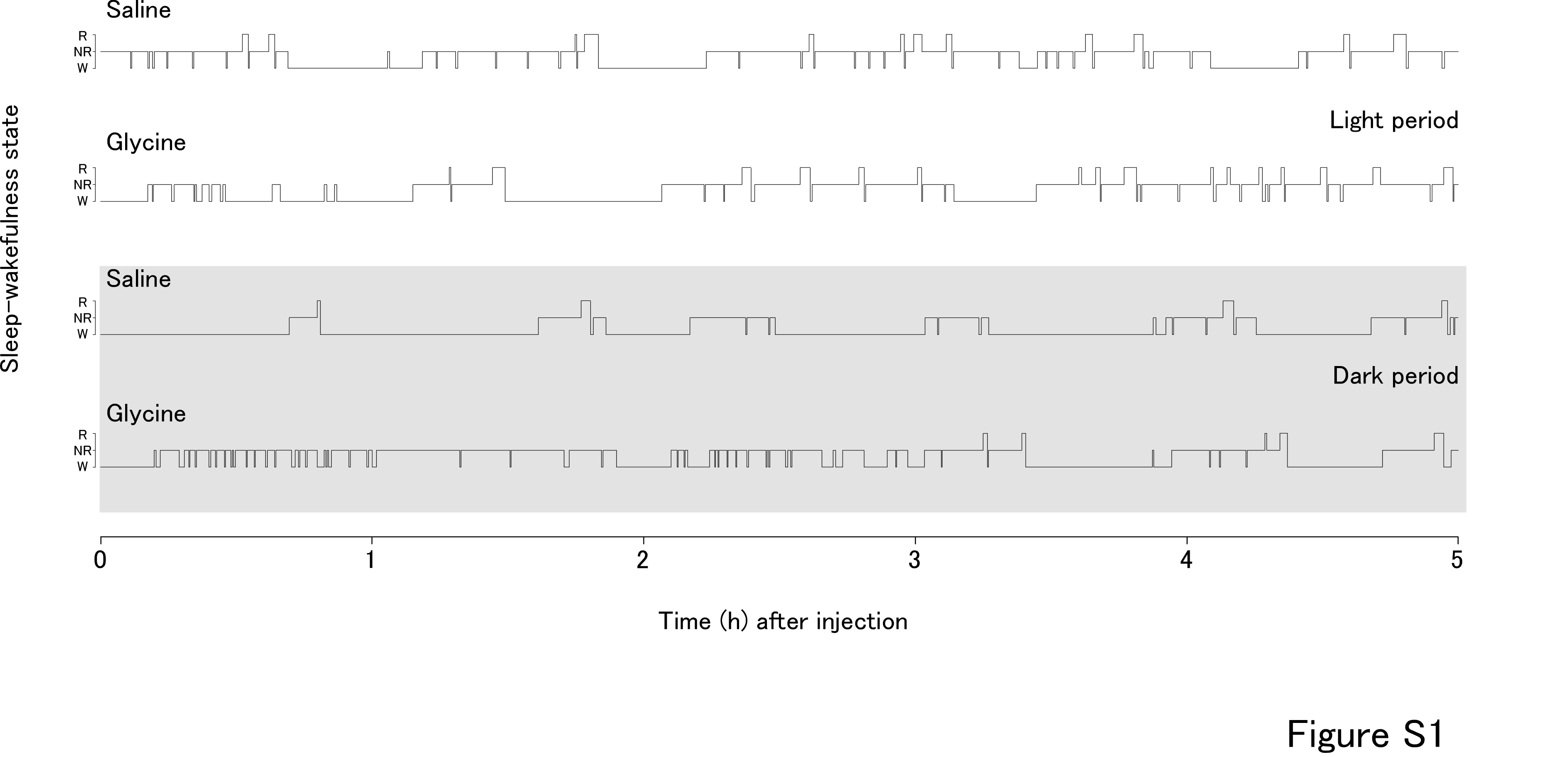

Supplement: Figure S1 — Representative 5 h dark/light period hypnograms for mice after saline and glycine administration. The shaded areas represent the dark period. W, awake; NR, non-rapid eye movement (REM) sleep; R, REM sleep. Glycine administered mice showed fragmentation of sleep/wake states in dark phase. (TIF) [file pone.0025076.s001.tif]

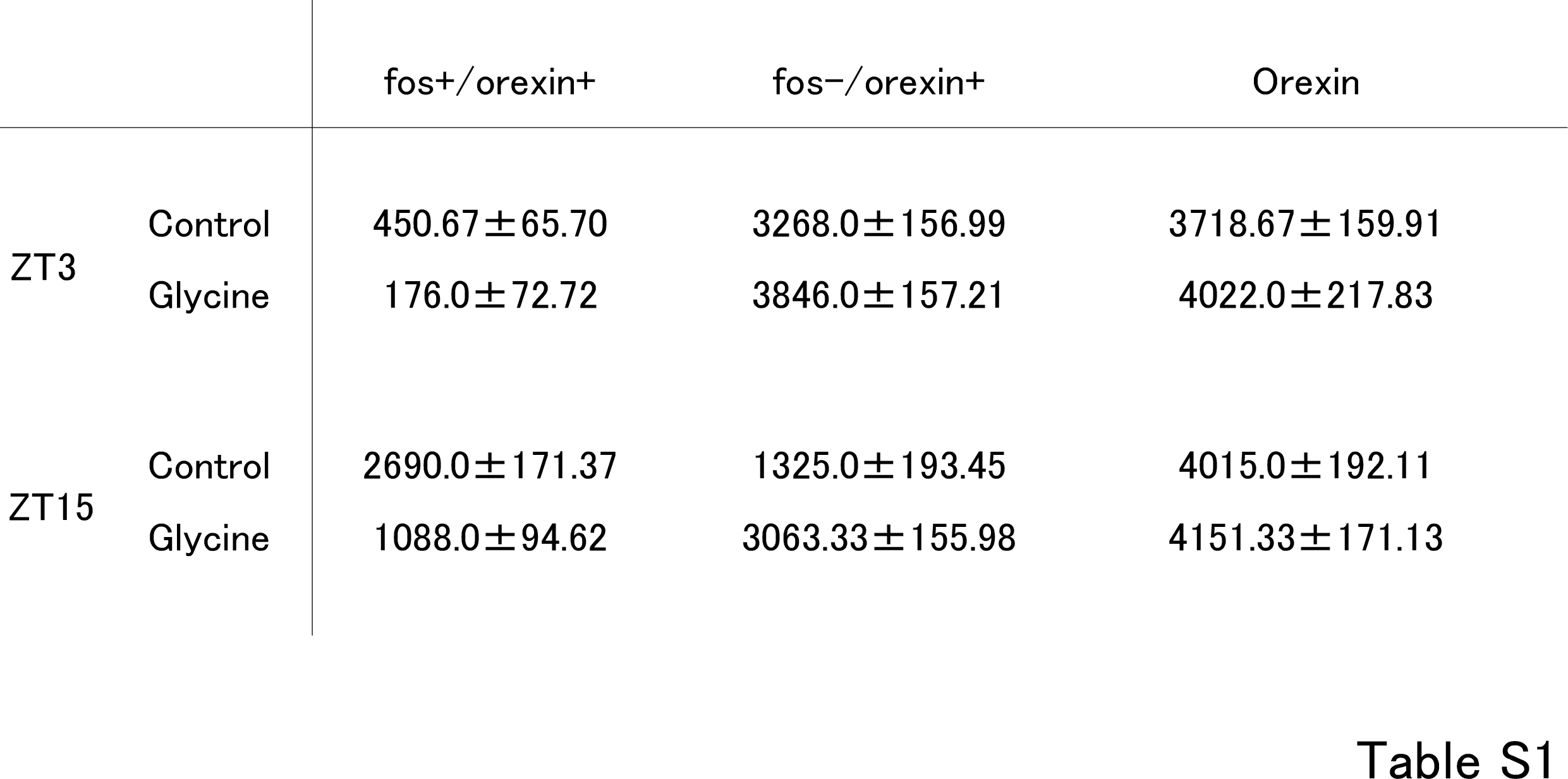

Supplement: Table S1 — Numbers of orexin neurons with or without Fos immunoreactivity in nuclei after glycine or saline administration. Mice were administered with glycine or saline at ZT0 or ZT12, and sacrificed for immunostaining at ZT3 or ZT15, respectively. (TIF) [file pone.0025076.s002.tif]
